# Protocol for induction and quantification of injury-primed axon outgrowth in adult mouse DRG neurons

**DOI:** 10.1016/j.xpro.2026.104755

**Published:** 2026-07-31

**Authors:** Eliana Seburn, Brett J. Hilton

**Affiliations:** 1 Department of Cellular and Physiological Sciences, University of British Columbia, Vancouver, BC V5Z 1M9, Canada; 2 International Collaboration on Repair Discoveries (ICORD), University of British Columbia, Vancouver, BC, Canada; 3 Djavad Mowafaghian Centre for Brain Health, University of British Columbia, Vancouver, BC, Canada

**Keywords:** Cell culture, Cell isolation, Neuroscience

## Abstract

Adult dorsal root ganglion (DRG) neurons provide a powerful model to study axon growth. Dissociated lumbar L3–L5 DRG neurons extend axons *de novo* in culture, enabling the measurement of baseline outgrowth. Here, we present a protocol for inducing and quantifying injury-primed axon outgrowth in adult mouse DRG neurons. We describe steps for sciatic nerve conditioning injury, isolating and culturing injured L3–L5 DRG neurons, and quantifying axon outgrowth. This protocol is compatible with cytoskeletal imaging and molecular profiling of injury-induced changes.

## Highlights

• Reproducible sciatic nerve “conditioning” lesion to induce injury-primed axon growth
• Isolation and culture of peripherally injured L3–L5 DRG neurons
• Quantitative analysis of axon outgrowth and branching in culture

## Abstract (editor highlights)

Publisher’s note: Undertaking any experimental protocol requires adherence to local institutional guidelines for laboratory safety and ethics.

## Graphical abstract

**Figure:**
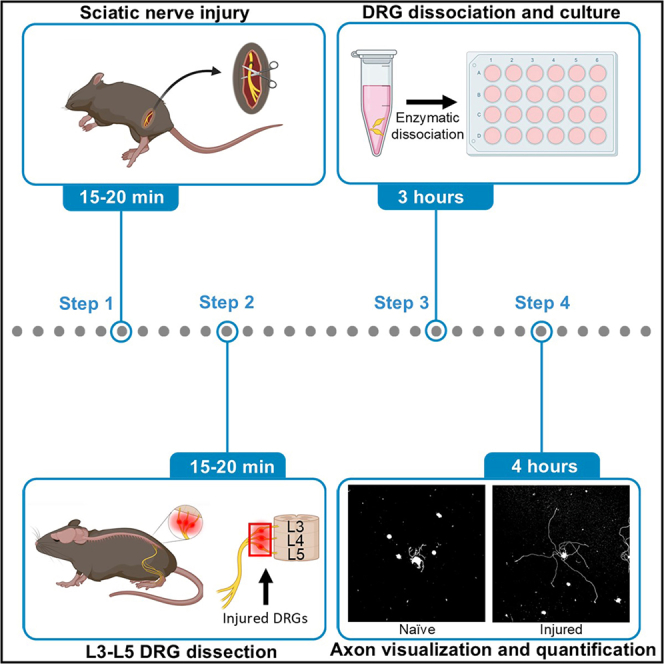

## Before you begin

Neurons in the adult central nervous system (CNS) are largely unable to regenerate their axons following injury.^1^^,^^2^^,^^3^ However, neurons in the peripheral nervous system (PNS) regenerate axons following injury.^4^ Dorsal root ganglia (DRG) neurons provide a powerful system to study these differences in regenerative capacity. DRG neurons are pseudounipolar and extend one branch into the CNS and another into the PNS from the same cell body. Injury to the peripheral branch conditions DRG neurons to enter a growth-permissive state, whereas injury to the central branch does not induce a regenerative response.^5^^,^^6^^,^^7^

The sciatic nerve contains peripheral axons of sensory neurons whose cell bodies reside in the lumbar L3, L4, and L5 DRGs.^8^ Whereas rat sciatic DRGs originate predominantly from L4 and L5, mouse DRGs predominantly originate from L3 and L4 and there are strain differences in the number of lumbar levels.^8^ Injury to the sciatic nerve distal to the DRG cell bodies induces transcriptional and cellular changes that enhance intrinsic axon growth capacity while reducing branching.^9^ Culture of L3-L5 DRGs enables analysis of morphological features, axon growth dynamics, and gene or protein expression changes that occur following peripheral nerve injury.^10^^,^^11^^,^^12^

In this protocol, we provide step-by-step instructions for performing a sciatic nerve injury to condition L3-L5 DRG neurons, followed by their isolation and culture to assess injury-induced changes in axon outgrowth.

### Innovation

Mechanisms of axon regeneration are frequently studied using DRG neurons, which can reacquire the ability to grow axons after injury. The conditioning injury paradigm is a widely used experimental approach to investigate these mechanisms. In this model, injury to the sciatic nerve enhances the intrinsic growth capacity of DRG neurons.

The protocol described here combines an *in vivo* conditioning injury with *ex vivo* neuron culture to evaluate the enhanced growth potential of DRG neurons following peripheral nerve injury. This approach provides an accessible framework to study cellular and molecular mechanisms that regulate axon growth. The protocol includes procedures for performing sciatic nerve injury, isolating and culturing peripherally axotomized DRG neurons, and visualizing and quantifying axon outgrowth in culture.

### Institutional permissions

The following protocol involves procedures on mice. These procedures were performed in accordance with the animal care guidelines of The University of British Columbia. The animal care protocol was reviewed and approved by The University of British Columbia Animal Care Committee in accordance with the guidelines of the Canadian Council on Animal Care. Experiments involving mice must be conducted in accordance with national and institutional guidelines and regulations.

### Surgical tool preparation

**Timing: 1 h, prepare day of surgery**

**Figure 1:**
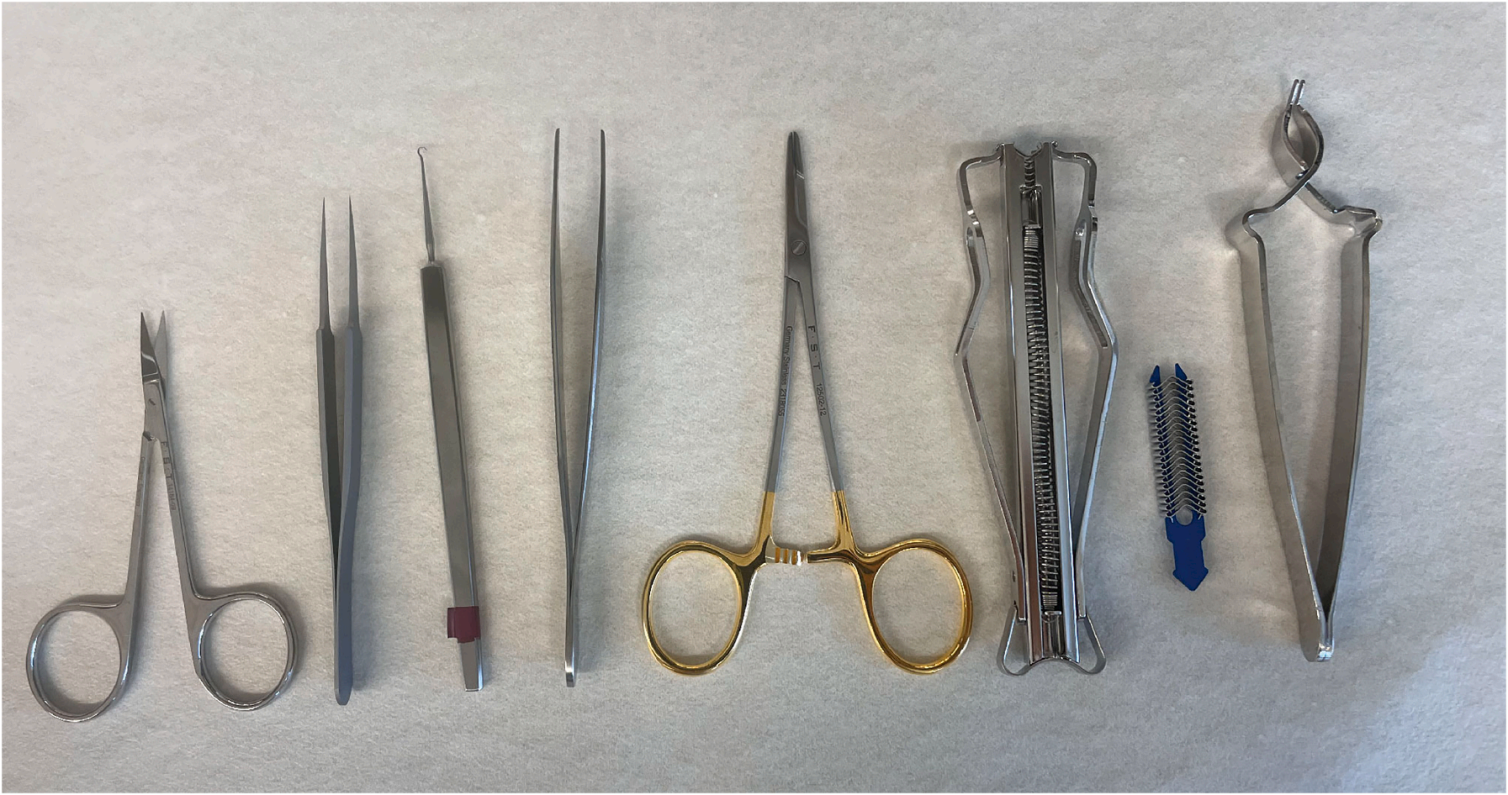
Surgical tools From left to right: Small iris scissors (FST: 14184-09), Dumont 5SF forceps (FST: 11252-00), Adson forceps (FST: 11006-12), Olsen-Hegar needle holder (FST: 12502-12), 9 mm Autoclip applicator (FST: 12020-09), 9 mm Autoclips (FST: 12022-09), Autoclip remover (FST: 12023-00).

1. Sterilize surgical instruments by autoclaving prior to surgery. Required instruments can be found in the materials and equipment section (Figure 1).

## Key resources table

**Table:** 

REAGENT or RESOURCE	SOURCE	IDENTIFIER
**Antibodies**		

Rabbit anti-βIII-tubulin (dilution 1:1000)	Sigma-Aldrich	Cat#: T2200, RRID:AB_262133
Alexa Fluor 488 AffiniPure Donkey Anti-Rabbit IgG (H+L) (dilution 1:1000)	Jackson ImmunoResearch Labs	Cat#: 711-545-152, RRID:AB_2313584
1 mg/mL DAPI (4′,6-Diamidino-2-Phenylindole, Dihydrochloride) (dilution 1:1000)	Fisher Scientific	PIEN62248

**Chemicals, peptides, and recombinant proteins**		

0.015 mg/mL Buprenorphine	Ceva	DIN: 02468085
Systane eye ointment	Systane	Cat#: 21667465_EA
0.9% sterile saline	ThermoFisher	Cat#: J67572.AP
Isoflurane	Abbott	Cat#: PZN 4831850
HBSS	ThermoFisher	Cat#: 14175095
HEPES	ThermoFisher	Cat#: 15630080
70% ethanol	VWR	Cat#: BDH1164-4LP
DMEM/F12	ThermoFisher	Cat#: 11330057
1.25% Collagenase IV	Millipore Sigma	Cat#: C1889
0.05% trypsin	ThermoFisher	Cat#: 25300054
Heat-inactivated fetal bovine serum	Fisher Scientific	Cat#: 16-140-071
1% penicillin/streptomycin	Fisher Scientific	Cat#: 15-140-122
L-Glutamine	ThermoFisher	Cat#: 25030081
B27 supplement	ThermoFisher	Cat#: 17504044
PBS	Sigma-Aldrich	Cat#: P7059
Laminin	Millipore Sigma	Cat#: L2020
Paraformaldehyde	Sigma-Aldrich	Cat#: 158127
Bovine serum albumin	Millipore Sigma	Cat#: A7906
Cold fish skin gelatin	Millipore Sigma	Cat#: G7765
Borax	Millipore Sigma	Cat#: 71996
Boric acid	Millipore Sigma	Cat#: B6768
NH4Cl	Sigma-Aldrich	Cat#: 213330
Triton X-100	Millipore Sigma	Cat#: X100
Fluoromount	Cedarlane	Cat#: 0100-01

**Experimental models: Organisms/strains**		

Mouse: C57Bl/6J, male and female, 8-12 weeks old	Jackson Laboratory	JAX:000664

**Software and algorithms**		

Fiji (ImageJ)	(Schindelin et. al.^13^)	https://imagej.net/software/fiji/downloads
MATLAB	Mathworks Inc.2022	https://www.mathworks.com/help/install/ug/install-products-with-internet-connection.html

**Other**		

Dumont 5SF forceps	FST	Cat#: 11252-00
0.3mm Graefe micro hook	FST	Cat#: 10062-12
Standard surgical scissors with stop	FST	Cat#: 14200-21
Small iris scissors	FST	Cat#: 14184-09
Olsen-Hegar needle holder	FST	Cat#: 12502-12
Adson forceps	FST	Cat#: 11006-12
9mm AutoClip applicator	FST	Cat#: 12020-09
9mm AutoClip clips	FST	Cat#: 12022-09
AutoClip remover	FST	Cat#: 12023-00
Microbead sterilizer	Fisher Scientific	Cat#: 14955340
Vannas-Tubingen spring scissors	FST	Cat#: 15005-08
Vannas spring scissors	FST	Cat#: 15018-10
5/0 monofilament nylon nonabsorbable sutures	MedRep Express	Cat#: 668B
Sterile surgical gauze	VWR	Cat#: CA53594-142
Sterile cotton-tipped applicator	ULINE	Cat#: 25-806 2WC
P2- Series Balance	VWR	Cat#: 76175-404
50 mL Falcon conical tube	Fisher Scientific	Cat#: 14-959-49A
15 mL Falcon conical tube	VWR	Cat#: CA21008-918
50 mL Steriflip vacuum tube top filter, 0.22 μm	Millipore Sigma	Cat#: SCGP00525
Nunc 24-well polystyrene treated culture plate	ThermoFisher	Cat#: 142475
Nunc 4-well polystyrene treated culture plate	ThermoFisher	Cat#: 176740
13 mm glass coverslips	Electron Microscopy Services	Cat#: 71861-026-C
2 mL microcentrifuge tubes	VWR	Cat#: 10025-738
1 mL syringe	VWR	Cat#: CABD309659
18G needle	VWR	Cat#: 76582-360
26G needle	VWR	Cat#: 76582-390
Razor blade	VWR	Cat#: 100491-866
70 μm cell strainer	VWR	Cat#: CA21008-952
Mini centrifuge, 115 V	VWR	Cat#: C0803
Heraeus Megafuge 16R centrifuge	Thermo Scientific	Cat#: 75004271
Stereomicroscope	Carl Zeiss	model: Stemi 508
Fluorescence microscope	Zeiss	model: Axio Imager.M2

## Materials and equipment

**Table:** Poly-L-Lysine

Reagent	Final concentration	Amount
Poly-L-Lysine	1 mg/mL	10 mg
Borate buffer	N/A	10 mL
**Total**	**N/A**	**10 mL**

***Note:*** Prepare fresh the day of the culture. Filter sterilize using 0.22 μm vacuum filter. Store at 4°C until use.

**Table:** Borate buffer, pH 8.5

Reagent	Final concentration	Amount
Boric acid	3.19 g/L	3.19 g
Borax	4.75 g/L	4.75 g
ddH2O	N/A	1 L
**Total**	**N/A**	**1 L**

***Note:*** Adjust pH to 8.5 using 1M NaOH as needed. Autoclave and store up to 6 months at 20°C.

**Table:** Laminin

Reagent	Final concentration	Amount
Laminin	5 μg/mL	50 μL
1X PBS	N/A	9.95 mL
**Total**	**N/A**	**10 mL**

***Note:*** Prepare fresh the day of the culture. Filter sterilize using 0.22 μm vacuum filter. Store at 4°C until use.

**Table:** DRG Collection Media

Reagent	Final concentration	Amount
HBSS −/−	N/A	14.9 mL
HEPES	1×	100 μL
**Total**	**N/A**	**15 mL**

***Note:*** Filter sterilize using 0.22 μm vacuum filter. Store at 4°C for up to 3 months.

**Table:** DRG Dissociation media

Reagent	Final concentration	Amount
Penicillin/Streptomycin	1%	500 μL
Heat-inactivated Fetal Bovine Serum	10%	5 mL
DMEM/F12	N/A	44.5 mL
**Total**	**N/A**	**50 mL**

***Note:*** Prepare fresh for each culture. Filter sterilize using 0.22 μm vacuum filter. Store at 4°C until use.

**Table:** Collagenase IV Solution

Reagent	Final concentration	Amount
Collagenase IV	0.125%	150 μL
DRG Dissociation Media	N/A	1.35 mL
**Total**	**N/A**	**1.5 mL**

***Note:*** Prepare fresh for each culture directly prior to dissociation.

**Table:** DRG Plating Media

Reagent	Final concentration	Amount
Penicillin/Streptomycin	1%	500 μL
L-glutamine	1%	500 μL
B27	1%	600 μL
DMEM/F12	N/A	48.4 mL
**Total**	**N/A**	**50 mL**

***Note:*** Prepare fresh for each culture. Filter sterilize using 0.22 μm vacuum filter. Store at 4°C until use. Heat to 37°C prior to plating.

**Table:** Blocking solution

Reagent	Final concentration	Amount
Heat-inactivated Fetal Bovine Serum	2%	1 mL
Bovine serum albumin	2%	1 g
50% Cold fish-skin gelatin	0.2%	0.2 mL
1× PBS	**N/A**	48.8 mL
**Total**	**N/A**	50 mL

***Note:*** Aliquot into 3 mL and store at −20°C up to six months.

Required surgical instruments (Figure 1):

• 5SF forceps.
• 13 mm Graefe nerve hook.
• Iris scissors.
• Olsen-Hegar needle holder.
• Adson forceps.
• AutoClip wound clips.
• AutoClip wound clip applicator.
• AutoClip wound clip remover.

**Figure 2:**
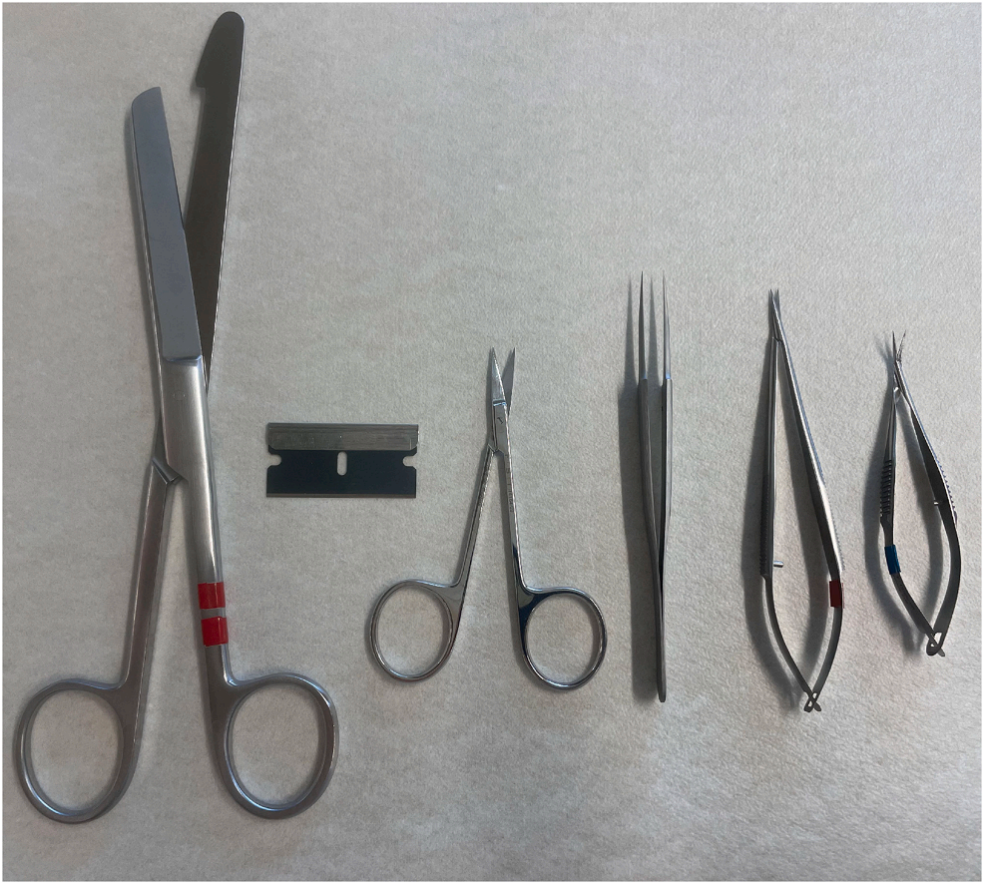
Dissection tools From left to right: Standard surgical scissors with stop (FST: 14200-21), razor blade (VWR: 100491-866), small iris scissors (FST: 14184-09), Dumont 5SF forceps (FST: 11252-00), Vannas spring scissors (FST: 15018-10), Vannas-Tubingen spring scissors (FST: 15005-08).

Required dissection instruments (Figure 2):

• Standard surgical scissors with stop.
• 5SF forceps.
• Vannas Tubingen spring scissors.
• Vannas spring scissors.
• Iris scissors.
• Razor blade.

## Step-by-step method details

### Sciatic nerve injury

**Timing: 15–20 min per mouse**

The purpose of this step is to injure the peripheral axon branch of DRG neurons to condition neurons for enhanced growth.

**Figure 3:**
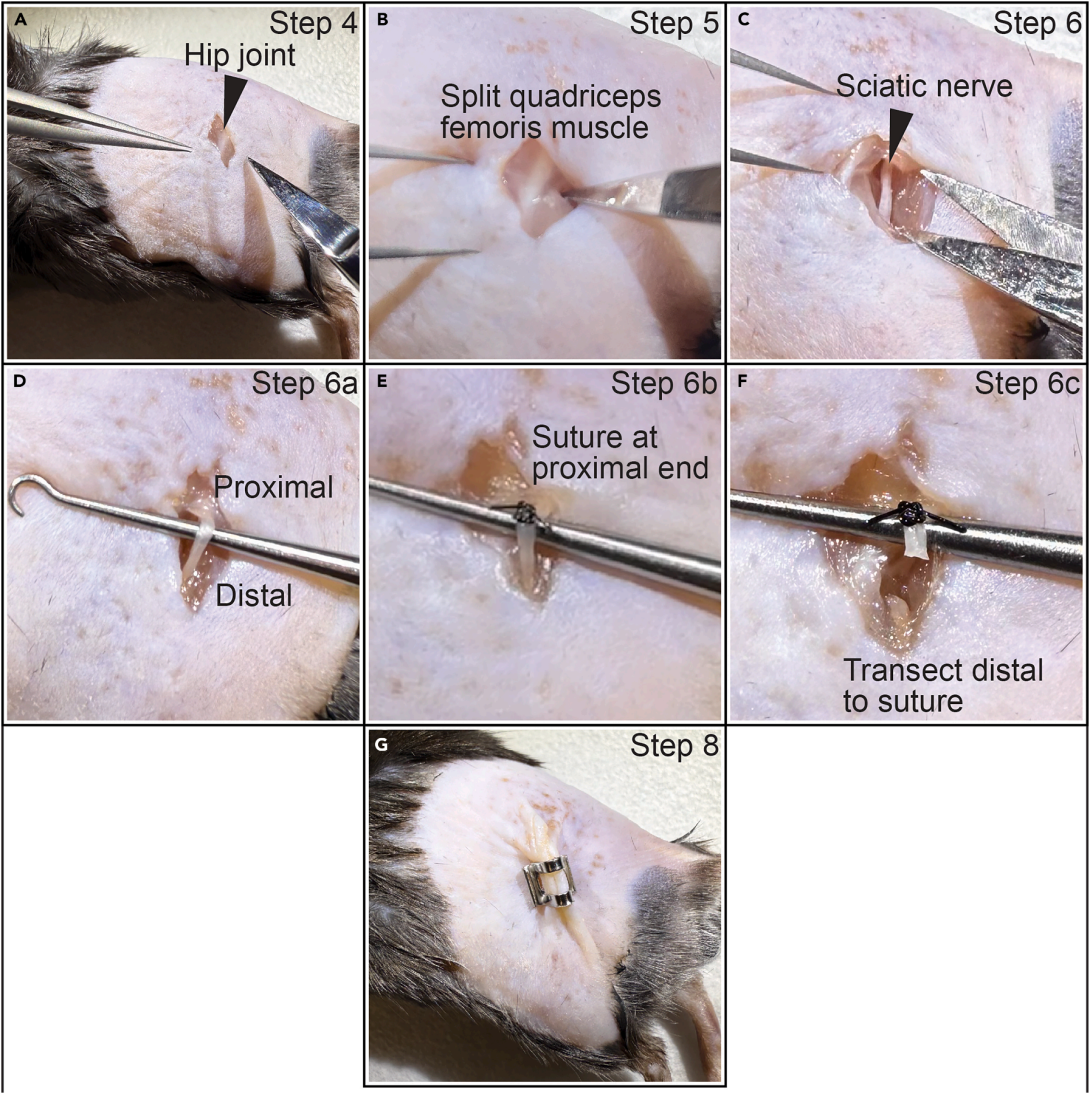
Sciatic nerve injury (A) 1 cm incision through the skin at the hip joint posterior to the femur bone. (B) Split the quadriceps femoris muscle posterior to the femur bone. (C) Spread open scissors to reveal the sciatic nerve. (D) Expose the sciatic nerve from the incision and let it rest on the Graefe hook. (E) Suture two knots around the proximal end of the sciatic nerve with 5/0 monofilament nylon non-absorbable sutures. (F) Transect the sciatic nerve 1 to 2 mm distal to the suture and place back into the leg. (G) Grab the skin around the incision and close with a wound clip.
5. Split the muscles on the thigh approximately 0.5 cm posterior to the hip joint, parallel to the femur bone by bluntly inserting the scissor tip 0.5 cm into the incision and gently opening the scissor tips to separate the muscle and reveal the sciatic nerve (Figure 3B).

***Note:*** Use blunt dissection and avoid cutting the muscle to prevent injury to the nearby blood vessel and accidental transection to the sciatic nerve.

***Note:*** The sciatic nerve can be identified as an off-white cord-like structure among the surrounding muscle. Lightly touching the sciatic nerve typically elicits a muscle twitch which can be used to confirm that you’ve identified the nerve correctly.

1. Anesthetize the mouse and prepare the surgical site.
  a. Weigh the mouse and record the weight. This will be used to calculate doses for analgesia and hydration support.
    ***Note:*** Recording and tracking the mouse’s weight prior to and following surgery is essential to monitor their health and recovery.
  b. Anesthetize the mouse under 5% isoflurane to a surgical plane of anesthesia using an induction chamber.
    **CRITICAL:** Test righting reflex, toe pinch reflex, and corneal reflex to ensure mouse has reached an adequate state of anesthesia prior to beginning surgery.
  c. Once a surgical plane of anesthesia has been reached, transfer the mouse to a nose cone and maintain surgical plane of anesthesia at 1-2% isoflurane.
  d. Apply Systane eye ointment using a sterile cotton tipped applicator.
  e. Inject weight appropriate doses of 0.9% sterile saline (20 mL/kg) and Buprenorphine (0.1 mg/kg) subcutaneously using a 26G needle.
    ***Note:*** Use an 18G needle to draw up 0.9% sterile saline to avoid bubbles in the syringe. Switch to a 26G needle for the injection.
  f. Shave the hindlimb from above the hip joint and down towards the femur and the tail.
    ***Note:*** Remove as much fur as possible during shaving. This reduces the risk of fur entering the incision site and causing infection.
  g. Clean the shaved area with sterile gauze soaked with chlorhexidine, followed by sterile gauze soaked with 70% ethanol. Repeat the chlorhexidine-ethanol cleansing cycle a total of three times.
2. Lay the mouse on its side with the surgical limb facing upward.
3. Assess surgical plane of anesthesia prior to beginning surgery.
  a. Pinch all four paws to test toe-pinch reflex and lightly touch eye to test corneal reflex. There should be an absence of each reflex when the mouse has reach a surgical plane of anesthesia.
4. Use forceps to lift the skin approximately 0.5 cm posterior to the hip joint and make a 0.5 cm incision with small Iris scissors (Figure 3A).

6. Spread open the incision with forceps to reveal the sciatic nerve (Figure 3C).
  a. While the incision is spread with forceps, use the micro hook to hook the sciatic nerve and gently pull the nerve out from the incision. Rest the nerve on the micro hook (Figure 3D).
    ***Optional:*** Sham controls for sciatic nerve injuries are widely used, as stretching and removal of the sciatic nerve, and inflammation from surgery, may minimally impact axon outgrowth. To incorporate a sham surgery into your experiment, proceed through steps 1-6a, including hooking and gently exteriorizing the sciatic nerve, but do not ligate or transect the nerve. Instead, skip steps 6b-6c and proceed directly to step 7 to return the intact nerve to the muscle cavity. Naïve controls are also acceptable and are used in this protocol.
    ***Optional:*** If a micro hook is not available, use a second pair of forceps to gently hook the nerve on one prong of the forceps to pull out the sciatic nerve. Lay the forceps on their side and let the nerve rest on one prong of the forceps.
  b. Make a double knot around the sciatic nerve at the proximal end of the exposed nerve with 5/0 monofilament nylon nonabsorbable sutures (Figure 3E).
    i. Trim the ends of the suture.
  c. Use small Iris scissors to completely transect the nerve ∼1-2 mm distal to the suture (Figure 3F). Transect the nerve at the mid-thigh level, proximal to the trifurcation of the sciatic nerve.
7. Use forceps to return the nerve to the muscle cavity.

**CRITICAL:** Maintain aseptic conditions by heat sterilizing tools for 20 s at 300°C in a bead sterilizer in between each mouse. Allow tools to cool down for 2–3 min prior to beginning the next surgery.

8. Grab the skin on either side of the incision with forceps and close the incision with a wound clip (Figure 3G).

**CRITICAL:** All steps following anesthesia must be performed on a heating pad set to 37°C to maintain core body temperature.

***Note:*** Mice can remove their sutures, and wound clips help to ensure their wound remains closed. These can remain on for one week. Mice should be lightly anesthetized to remove wound clips. Sutures can also be used to close the wound. However, for the purposes of this protocol, experimental endpoint is three days post-injury and wound clips can remain on until experimental endpoint.

### Coat coverslips

**Timing: 3 h, day of dissections**

9. Prepare cell culture media and reagents (see materials and equipment section for preparation details).

***Note:*** Prep enough coverslips to have 2 to 3 coverslips per mouse to have substantial technical replicates for quantification.

10. Coverslip preparation.
  a. Place 13 mm glass coverslips into each well of a cell culture plate.
    i. Wash once with 70% ethanol.
    ii. Aspirate the ethanol and wash three times with 1× PBS.
  b. Incubate each glass coverslip in poly-L-lysine (PLL) in 0.1 M borate buffer, pH 8.5 (1 mg/mL) for 1 h at 37°C.
    i. Wash each coverslip in 1X PBS three times and then leave to dry for 90 min in the biosafety cabinet.
      ***Note:*** Perform dissections while coverslips are drying after PLL coating.
  c. Incubate each glass coverslip in laminin (5 μg/mL in 1×PBS) for 1 h at 37°C. For a 24-well plate, 500 μL is sufficient to cover each coverslip.
    ***Note:*** Laminin incubation can occur during the Collagenase IV step of the culture protocol (Step 20).
    i. Wash each coverslip three times in 1X PBS.
11. Incubate coverslips in DMEM/F12 at 37°C until plating.

***Note:*** Prepare two to three 13 mm glass coverslips per mouse for unilateral sciatic nerve injuries. Prepare one coverslip per well for 4-well or 24-well plates with 1.9 sq. cm of culture area. This allows for adequate quantification of axon outgrowth as neurons are not growing at a density where their individual axons cannot be distinguished.

### Isolation of L3–L5 DRGs

**Timing: 15–20 min per mouse**

To assess the conditioning effect, it is only necessary to dissect L3-L5 DRGs. Alternate experimental endpoints are appropriate based on your experimental design. However, to assess the enhancement of axon outgrowth in culture following peripheral axotomy, three days post-injury is optimal.

***Note:*** This protocol is optimized for C57/Bl6 mice. Vertebral number can vary between mouse strain/substrain. Therefore, lumbar DRG identification should rely on consistent anatomical landmarks rather than assuming vertebral level maps identically across all mouse backgrounds.

12. Assemble dissection instruments. Required instruments can be found in materials and equipment section (Figure 2).
13. Prior to euthanasia, prepare ice-cold HBSS + HEPES solution in 2 mL microcentrifuge tubes, one per sample.
14. Euthanize the animal in accordance with the approved protocol.
  a. At three days post-injury, anesthetize the mouse with 5% isoflurane to a deep plane of anesthesia. After the mouse reaches a deep plane of anesthesia, swiftly decapitate the mouse using large surgical scissors.
  b. Transfer the mouse to ice immediately following euthanasia.

**CRITICAL:** Minimize the time between euthanasia and dissection to preserve cell viability.

15. Perform a laminectomy and remove the spinal cord to locate DRGs.
  a. Place the mouse ventral side down on a wax dissecting tray and pin down each foot to stabilize the mouse. Soak the back of the mouse with 70% ethanol.
  b. With a sharp razor blade, make a midline incision through the skin above the vertebral column from the cervical section of the vertebral column to the base of the tail (Figure 4A).
    i. Separate the skin from the underlying muscle and tissue to make the vertebral column more accessible (Figure 4B).
      **CRITICAL:** Avoid getting fur on the tissue covering the vertebral column to avoid fungal contamination in culture.

**Figure 4:**
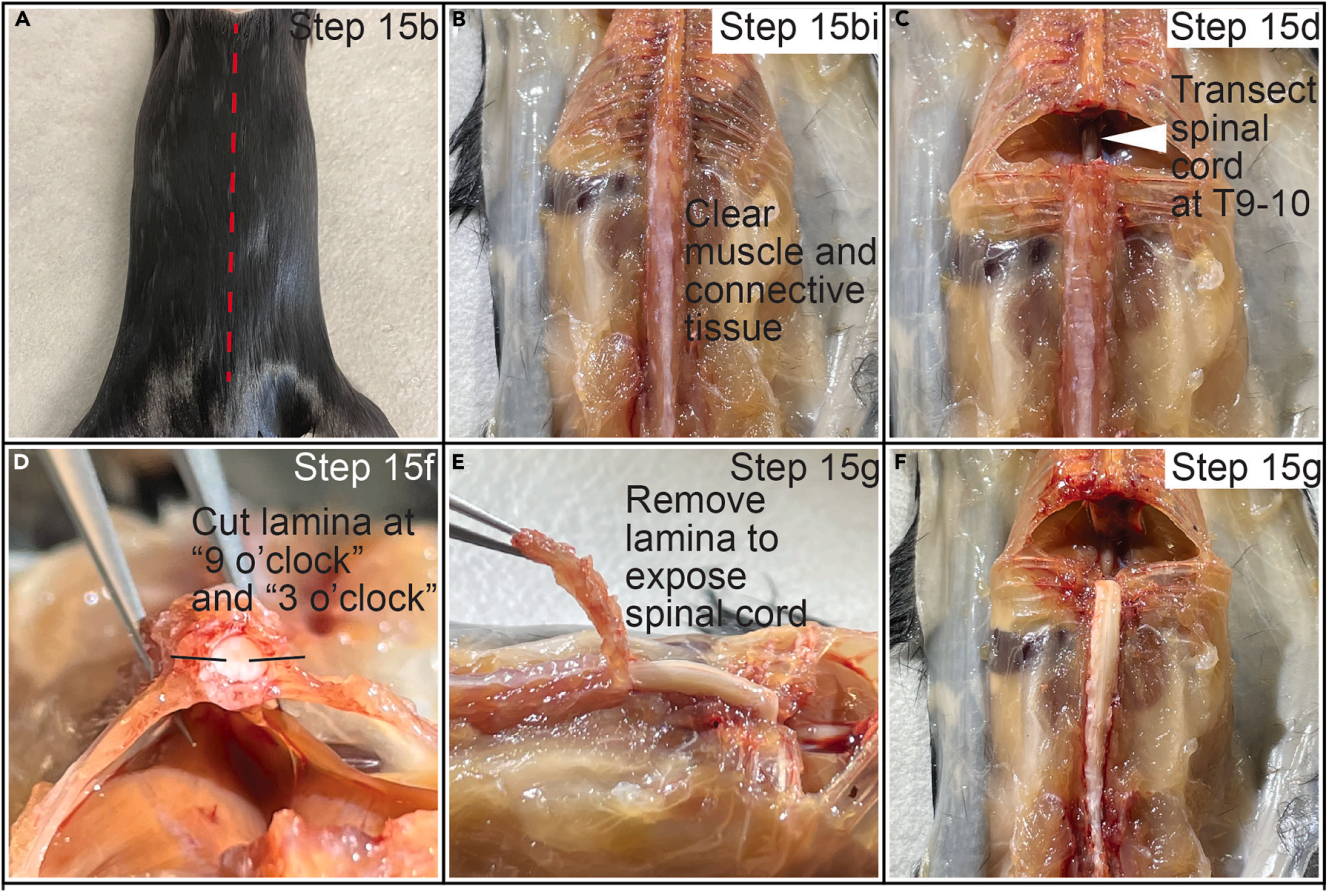
Laminectomy (A) Lay the mouse on its ventral side and make a midline incision with a razor blade above the vertebral column to spread open the skin. Red line represents incision. (B) Clear the muscle and connective tissue surround the vertebral column. (C) Transect the vertebral column at T9-T10 to reveal a cross-section of the spinal cord for the laminectomy. White arrow represents transection site. (D) Insert a pair of spring scissors between the vertebrae and the spinal cord and make cuts at “9 o’clock” and “3 o’clock”. Black lines represent cutting sites. (E) Move caudally down the vertebral column making cuts at each vertebrae to remove the lamina and expose the spinal cord. (F) View of the exposed spinal cord.
  c. Cut the muscle and tissue on either side of the vertebral column with a razor blade to separate the tissue from the vertebral column.
  d. Transect the vertebral column at the T9-T10 level using small Iris scissors (Figure 4C).
  e. For the laminectomy use a pair of spring scissors that can cut through bone but are small enough to fit between the vertebrae and the spinal cord. We recommend 4 mm Vannas spring scissors.
  f. Grasp both sides of the vertebral column with forceps to stabilize the column. Use spring scissors and gently insert the tip into the space between the vertebrae and the spinal cord where the column has been transected (Figure 4D).
    ***Note:*** Imagining the vertebral column as a clock, where 12 o’clock is the dorsal surface, insert the spring scissors and make a cut at approximately 3 o’clock, then 9 o’clock, to sequentially remove the lamina of each vertebra (Figure 4D).
  g. Moving down the spine, grab the section of lamina that has been cut and gently pull it back from the spinal cord to reveal the next section for removal. Continue this process down the spine, stopping at the sacral vertebrae (Figures 4E and 4F).
16. Remove the spinal cord.
  a. Starting at the thoracic end of the spinal cord, grab the spinal cord with forceps and gently pull it out of the column.
    i. As the cord is removed, use a pair of spring scissors to cut away the central branches so as not to tug any of the DRGs out of the column with the spinal cord. DRGs remain embedded in the intervertebral foramina.
17. Remove L3–L5 DRGs.
  a. Once the spinal cord is removed the DRGs and their attached central branch should be visible in the intervertebral foramen. DRGs are round and more translucent than their attached nerves (Figure 5A).

**Figure 5:**
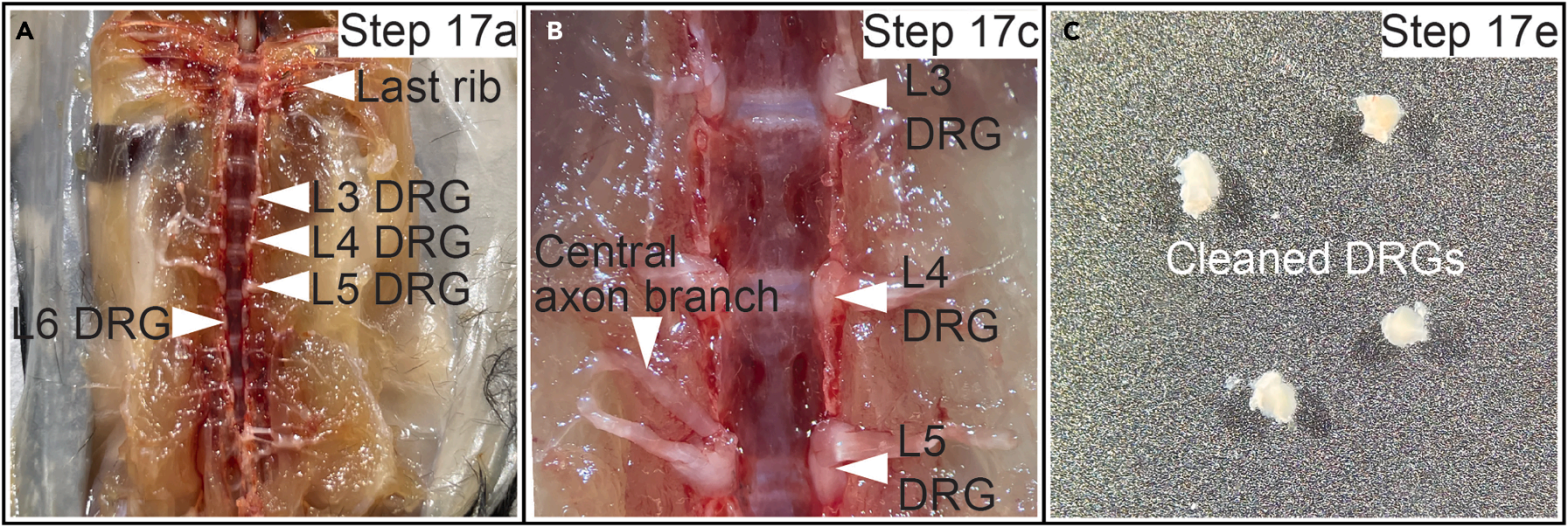
L3-L5 DRG dissection (A) View of the vertebral column with the spinal cord removed showing DRGs visible at each intervertebral foramen. Arrows indicate to the last rib, and L3, L4, and L5 DRGs, and the smaller L6 DRG. (B) Close-up view of the L3, L4, and L5 DRGs with their attached central axon branch. Arrows indicate the L3, L4, and L5 DRGs and the central axon branch of one L5 DRG. (C) Dissected L4 and L5 DRGs with both central and peripheral axon branches removed.
  b. To identify the L3-L5 DRGs, locate the most caudal rib, which marks the T13 vertebral segment. The DRG located at this segment is the T13 DRG. Count caudally from the T13 DRG to identify the L3, L4, and L5 DRGs. L4 is typically the largest DRG whereas L6 serves as a caudal landmark because it is much smaller than L5 (Figure 5A).
  c. To remove each DRG, gently grasp the central axon branch and slightly pull each DRG out of the intervertebral foramen (Figure 5B).
    i. The DRGs may be under a thin layer of meninges. Cut off the meninges surrounding the DRG that may be holding it in place.
  d. Cut off the descending branch of the nerve to further remove the DRG from the intervertebral foramen, until it is only attached to its central branch.
  e. To reduce myelin debris and contamination, remove as much of the central and peripheral branch of the nerve until only the DRG remains (Figure 5C).
    **CRITICAL:** Neuronal cell bodies reside within the DRG. Excessive force and handling can damage the neuronal cell bodies and impact cell viability. Avoid grasping the DRG directly; instead, use the central axon branch to manipulate its position. When the DRG must be picked up, scoop it with the tips of the forceps rather than pinching it.
  f. Place dissected DRGs into a 2 mL Eppendorf tube with ice-cold HBSS-HEPES.

### DRG neuron culture

**Timing: 3 h**

Injured DRG neurons retain their capacity for enhanced axon growth *in vitro*. L4-L5 DRG neurons can be cultured for 12–16 h to assess this difference in axon outgrowth.

***Note:*** The following steps should be performed in a biosafety cabinet. Spray down the interior of the biosafety cabinet, and any materials that enter the biosafety cabinet, with 70% ethanol prior to use. Use sterile pipette tips.

18. Spin down the DRGs at 2000 × *g* for 15 s in a 2 mL Eppendorf tube to collect the DRGs at the bottom of the tube.
19. Aspirate the HBSS-HEPES. Take care to avoid aspirating the DRGs from the bottom of the tube as they do not form a firm pellet when spun down. Using a pipette for these steps minimizes the risk of aspirating the DRGs.
20. Incubate DRGs in collagenase IV (0.125%) at 37°C for 90 min.
21. Spin down at 2000 × *g* for 15 s and wash with DMEM/F12. Repeat for a total of three washes.
22. Incubate DRGs in 0.05% trypsin-EDTA for 10 min at 37°C.
23. Spin down at 2000 × *g* for 15 s and wash with dissociation media (DMEM/F12 with 10% FBS and 1% Pen/Strep). Repeat for a total of three washes.
24. Resuspend the ganglia in dissociation media for mechanical dissociation.
  a. Pipette up and down with a p1000 50–60 times to mechanically dissociate DRGs into a cell suspension. Mechanical dissociation is complete when the cell suspension appears cloudy with no chunks of tissue remaining.
25. Place a 70 μm nylon cell strainer on a 50 mL Falcon tube and pre-wet with 1 mL dissociation media.
26. Filter the single cell suspension through the cell strainer.
  a. Fill the 2 mL Eppendorf tube once more with 1 mL of dissociation media to wash the remaining cells out of the tube. Filter this wash through the nylon cell strainer.
27. Centrifuge the filtered cells at 300 × *g* for 10 min.
28. Locate the pellet at the bottom of the 50 mL Falcon tube. Aspirate the dissociation media, taking care to avoid disrupting the pellet.
29. Resuspend the cell pellet in plating media (DMEM/F12 with 1% penicillin/streptomycin, B27 supplement, and L-glutamine). Pipette up and down 5-6 times to ensure there are no clumps left after resuspending.
30. Using a hemocytometer, count and adjust the concentration of cells to an appropriate dilution.
  a. Combine 5 μl of cell suspension with 5 μl of Trypan Blue and load onto the hemocytometer. Count and record the number of unstained (live) cells in each quadrant.
  b. Estimate cells/mL using the following equation: Cells/mL = (total neurons counted in 4 quadrants) × 2 (dilution factor) × 10,000 (hemocytometer volume conversion factor).
  c. Adjust the cell suspension to 1000 neurons per 500 mL of plating media.

***Note:*** Resuspend the cells in an appropriate quantity of plating media for the desired plating density for axon outgrowth quantification. The typical neuronal yield is ∼30,000 from the pool of 6 DRGs (lumbar L3–L5 bilaterally) or ∼5,000 neurons per DRG. We recommend 500 μL of plating media per well of a 24-well plate, and plating at a density of 1000 neurons per 500 μL. This is the recommended density to allow for accurate axon outgrowth quantitation, as it allows neurons to extend their axons without overlapping.

31. For a 24-well plate, add 500 μL of cells resuspended in plating media to each well to plate 1000 neurons per well.

***Optional:*** Cell debris and glia can be reduced in the culture using enrichment methods such as bovine serum albumin (BSA) or Percoll gradients. However, this step is not necessary to quantify axon outgrowth in this context.

32. Incubate cells for 12–16 h at 37°C and 5% CO2.

***Note:*** The sciatic nerve injury potentiates axon growth in culture. 12-16 h in culture allows the naïve condition to sprout neurites, while minimizing excessive neurite overlap that complicates quantification.

### Immunocytochemistry

**Timing: 4 h**

33. After 12–16 h in culture, fix the cells in room temperature 4% PFA with 4% sucrose in 1X PBS.
  a. Aspirate the existing culture media and replace with room temperature 4% PFA with 4% sucrose in 1× PBS for 15 min at 20°C. Wash three times with 1× PBS.

***Note:*** All suggested volumes are for 4-well or 24-well plates with 1.9 sq. cm of culture area.

***Optional:*** To avoid aspirating cells, add 16% PFA with 16% sucrose to the existing culture media to a concentration of 4% PFA and 4% sucrose, then aspirate the culture media and wash three times with 1× PBS.

***Note:*** Avoid pipetting directly onto the coverslip. Pipette along the side of the well to avoid damaging or detaching neurons.

***Optional:*** The protocol can be paused after the final wash with 1× PBS before antibody staining, with coverslips stored in the fridge at 4°C for up to 8 weeks.

34. Quench cells for 10 min in 500 μL 50 mM NH_4_Cl.
35. Wash three times with 500 μL 1× PBS.
36. Permeabilize cells with 500 μL of 0.1% Triton X-100 in 1× PBS for three min.
37. Block cells and incubate with primary antibodies.
  a. Incubate cells in 200 μL of 100% blocking solution (2% fetal bovine serum, 2% bovine serum albumin, and 0.2% cold fish skin gelatin in 1× PBS) for 1 hour at 20°C.
    ***Note:*** Blocking and antibody staining can also be performed at 4°C overnight. The proposed conditions are optimized for our culture conditions and may be specific to the antibodies used in our lab. Other antibodies may be used but conditions will have to be optimized for each antibody. To visualize axon outgrowth, we suggest anti-βIII-tubulin.
    ***Note:*** For a 24-well plate, 200 μL of blocking or antibody solution is sufficient to fully cover the coverslip.
    ***Note:*** To visualize axon growth, we use rabbit anti βIII-tubulin (1:1000), DAPI (1:1000) and donkey anti-rabbit AlexaFluor488 (1:1000).
  b. Incubate cells in 200 μL of primary antibody diluted into 1X PBS with 10% blocking solution for 1 hour at 20°C.
    i. For negative controls, incubate cells in 1X PBS with 10% blocking solution for 1 hour at 20°C with no primary antibody added. Proceed with the following steps as written.
      ***Note:*** Ensure there are enough coverslips to test proper negative controls for antibody staining.
  c. Wash cells four times with 500 μL of 1× PBS.
38. Incubate cells with secondary antibodies.
  a. Incubate coverslips in 200 μL of secondary antibody solution in 1X PBS with 10% blocking solution for 1 hour at 20°C.
  b. Wash cells four times with 500 μL of 1× PBS, then once in 500 μL ddH2O.
39. Mount the coverslips onto glass microscope slides.
  a. Place ∼15 μL of Fluoromount on a glass slide for each coverslip. Up to 4 coverslips can be mounted on one slide.
  b. Remove the coverslips from each well using a pair of forceps. Let the water drip off the side of the coverslip.

***Note:*** Touch only the very edges of the coverslip with the forceps. Scraping the cell side of the coverslip will scratch cells and axons off the coverslip.

***Note:*** These forceps should be separate from dissection and surgery tools, as the tips of the forceps may get damaged when touching plastic and glass.

40. Gently place the edge of coverslip cell side down on to the drop of Fluoromount, until it forms a seal with the edge of the coverslip. Then, fully drop the coverslip onto the Fluoromount.
41. Gently press the middle of the coverslip to ensure it lays flat on the slide.
42. Cure for at least 1 h at 20°C before imaging.

**Pause point:** Mounted coverslips can be stored at 4°C for up to 1 month protected from light before imaging. For fluorescence intensity comparisons, image all coverslips from the same experiment using the same microscope settings, and when possible, during the same imaging session. Avoid repeated imaging at different time points, as fluorescence intensity may decrease over time and with repeated light exposure. Follow instructions for recommended storage conditions from the manufacturer.

43. Acquire images of neurons using a 10× objective on a standard fluorescence microscope.

## Expected outcomes

**Figure 6:**
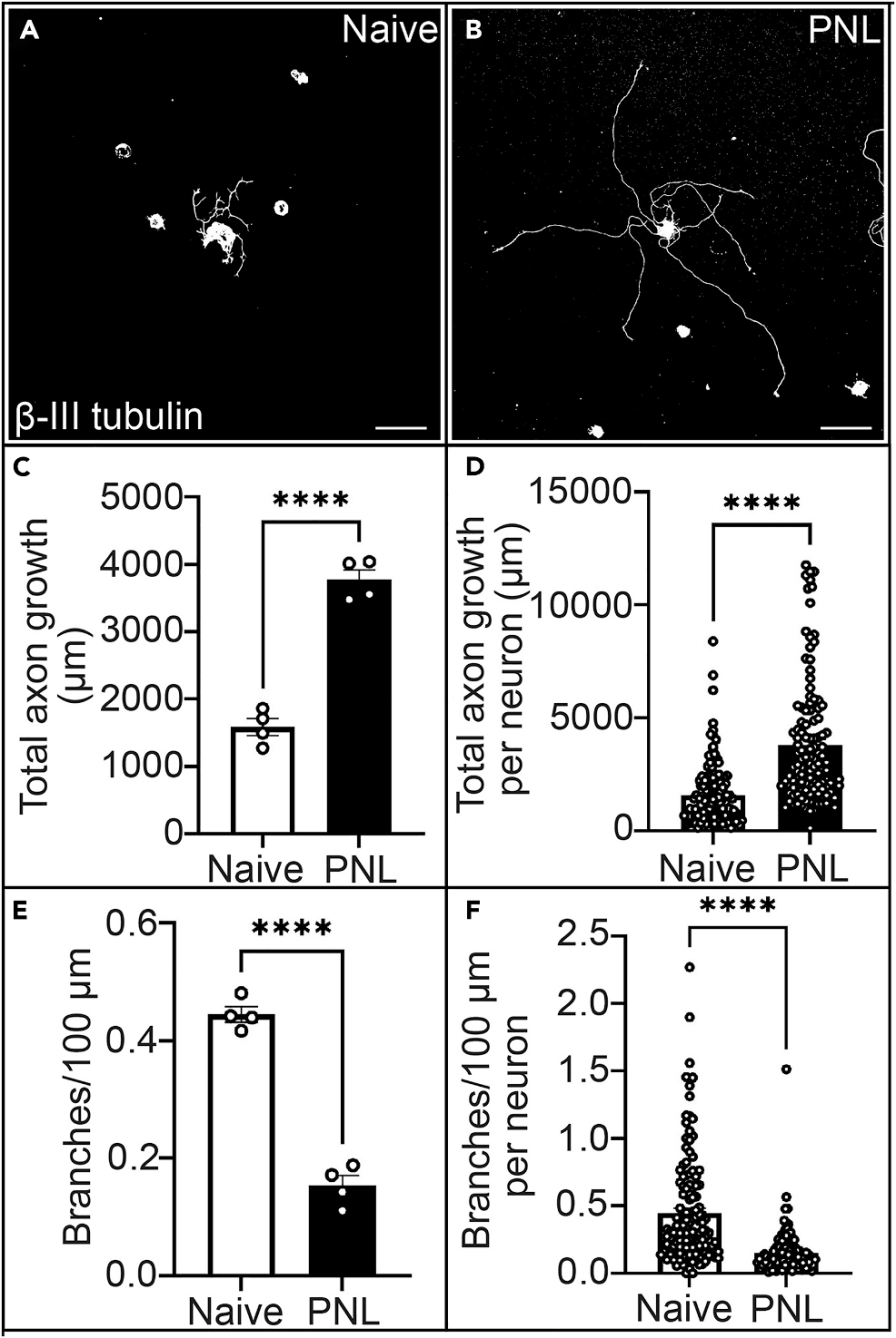
Representative cultures (A) Representative image of βIII-tubulin immunofluorescence of a naïve DRG neuron at 12 h in culture. Scale bar = 100 μm. (B) Representative image of βIII-tubulin immunofluorescence of an injured DRG neuron at 12 h in culture. Scale bar = 100 μm. (C) Quantification of average total axon growth by biological replicate. *N* = 4 independent experiments. Values are plotted as mean ± SEM. ∗∗∗∗*p* < 0.0001 by Student’s *t* test. (D) Quantification of total axon growth per neuron. Individual neuron values are plotted. *N* = 133 naïve neurons and 119 PNL neurons. Horizontal bars indicate mean ± SEM. ∗∗∗∗*p* < 0.0001 by Student’s *t* test. (E) Quantification of branching/100 μm by biological replicate. *N* = 4 independent experiments. Values are plotted as mean ± SEM. ∗∗∗∗*p* < 0.0001 by Student’s *t* test. (F) Quantification of branching/100 μm per neuron. Individual neuron values are plotted. *N* = 133 naïve neurons and 119 PNL neurons. ∗∗∗∗*p* < 0.0001 by Student’s *t* test. Horizontal bars indicate mean ± SEM. ∗∗∗∗*p* < 0.0001 by Student’s *t* test.

This protocol details procedures for sciatic nerve injury and assess the subsequent effects on axon growth in cultured DRG neurons. Typical yields range from ∼3,000-6,000 neurons per DRG, depending on dissociation efficiency, animal age, and the time between euthanasia and dissection. At 12-16 h in culture, neurons isolated from injured DRGs extend long, sparsely branched axons, whereas neurons from naïve DRGs exhibit shorter, highly branches axons. This timepoint allows differences in axon growth to emerge while minimizing excessive axon overlap, which can complicate analysis. Quantitative analysis generally reveals a ∼1.5–2 fold increase in axon length in neurons derived from injured DRGs accompanied by a 2-fold reduction in branching (Figure 6).

## Quantification and statistical analysis

### Axon outgrowth quantification

**Timing: Variable (depending on number of neurons)**

***Note:*** The following steps are described for micrographs of individual neurons. However, neurons can be isolated for quantification from larger tile scans to reduce imaging time.

***Note:*** It is important to quantify at least 20–30 neurons per mouse/biological replicate. Neurons obtained from the same mouse represent technical replicates and should be averaged to correspond with their biological replicate for statistical analysis. Individual neurons form the same mouse should not be treated as separate biological replicates.

***Note:*** Only isolated neurons with clearly identifiable somata and axons should be included in the analysis. Neurons with overlapping axons or contacting neighboring cells should be excluded to ensure accurate quantification.

1. In ImageJ, 8-bit each image file and adjust the brightness to increase the contrast between the background and the neuron.
  a. Image→Type→8-bit. The image should be grayscale after this adjustment.
  b. Image→Adjust→Brightness and contrast. Adjust these setting until the contrast between the neuron and the background is satisfactory. Ensure the brightness adjustments remain the same across images.
2. Isolate the neuron and soma using the ImageJ Freehand selection tool.
  a. For each image, outline the entire neuron with the Freehand selection tool. Once the neuron is outlined, clear the background by Edit → Clear Outside and save the file with the prefix *neuronal-.*
  b. Duplicate the neuronal image.
  c. In the duplicated image, outline the soma using the freehand selection tool and clear the axons using Edit → Clear Outside.
  d. Apply a threshold to the soma using Image → Adjust → Threshold. Adjust the threshold until the whole soma is highlighted. Apply the threshold and save the image with the prefix *soma-.*
  e. Repeat for each neuron.
3. Save each file into a subfolder for each neuron. Each neuronal subfolder should contain one neuronal-file and one soma-file with identical suffixes.
4. For axon tracing, use the NeuriteTracer ImageJ^14^ macro to skeletonize and trace each neuron and trace its axons. The input file path leads to a main experiment folder, which contains subfolders organized first by experimental condition, and then by individual neuron. NeuriteTracer outputs traces into the original folders.
5. For axon outgrowth quantification, use the ShollAnalysis ImageJ^15^ macro. The input file path is identical to the NeuriteTracer macro. This macro completes a Sholl analysis for each individual neuron and outputs an Excel file with the data into the individual neuron folder.
6. Use MatLab to compile the Sholl analysis files from each neuron into a single Excel file. We suggest using GraphPad Prism as a graphing and analysis tool.

***Note:*** Axon growth can be quantified using metrics derived from Sholl analysis, including maximum neurite radius, total neurite length, or the number of Sholl intersections.

## Limitations

DRGs contain heterogeneous neuronal cell populations.^16^ Although many neurons that project through the sciatic nerve reside in L3-L5 DRGs, these ganglia also contain sensory neurons that project to other peripheral targets.^8^ As a result, not all neurons present in L3-L5 DRGs will be axotomized by sciatic nerve transection. Consequently, some neurons observed in the injured condition may exhibit little or no axon outgrowth, as they may not project through the sciatic nerve and therefore will not be injured. Furthermore, it can be difficult to standardize the lesion location, and more proximal and distal lesions can induce various levels of conditioning. Excessive stretching or manipulation of the nerve may also cause additional injury and impact the conditioning response. Handle the nerve gently, and avoid pulling or lifting the nerve under tension.

Additionally, cells are filtered through a 70 μm nylon cell filter, which may eliminate large diameter proprioceptive and mechanoreceptive neurons. For the purposes of this protocol, this should not interfere with axon outgrowth assays. However, it should be noted that the cells in culture may not be representative of native proportions of DRG neuron subtypes.

This protocol does not detail neuronal enrichment, but common enrichment protocols can be incorporated into this protocol to reduce debris and non-neuronal cells in the culture. Enrichment steps to eliminate debris and non-neuronal cells may impact the extent of axon outgrowth. However, the conditioning effect can still be detected after enrichment.

## Troubleshooting

### Problem 1: Neuronal cell loss

Dissociation of the DRG into a single-cell suspension culminates in loss of many neurons. Neuronal loss may be detected after plating by examining cultures under a brightfield microscope to assess cell recovery. Neuronal somas are larger than other cells present in the culture and should be readily visible at 10x magnification. Several factors can contribute to neuronal loss. Mechanical damage during DRG dissection may reduce survival, particularly if dissections are prolonged or performed long after the animal is euthanized. Excessive enzymatic dissociation time or elevated incubation temperatures during dissociation can also compromise neuronal viability. Additionally, sharp changes in temperature during the dissociation process may negatively affect cell survival. Neuronal loss can also occur during fixation if culture media is aspirated too aggressively from the wells.

### Potential solution

• Dissection efficiency improves with practice. Perform practice dissections and cultures prior to conducting critical experiments.
• Ensure all media for washes and dissociation are pre-warmed to 37°C before contacting the DRGs. Keep media in a 37°C bead bath during the day of culture.
• For fixation, avoid aspirating culture media from the wells. Instead, add 16% PFA + 16% sucrose to the culture medium to achieve a final concentration of 4% PFA. This approach minimizes cell loss during fixation (Step 33).

### Problem 2: Difficulty locating DRGs during dissection

DRGs are small structures and can be difficult to identify during vertebral column dissection. Proper visualization requires the use of a stereomicroscope and an adequate laminectomy. If cuts are made too deep or too ventrally, the DRGs may be damaged or inadvertently removed. Conversely, if cuts are made too shallow, the DRGs may remain partially embedded with the intravertebral foramen, making them difficult to visualize and more prone to damage during subsequent dissection.

### Potential solution

• Perform laminectomy cuts along the lateral edges of the dorsal vertebral column. If the dorsal surface of the vertebral column is considered the “12 o’clock” position, make cuts approximately at the “3 and 9 o’clock” positions to expose the DRGs while minimizing the risk of damaging them (Step 15F).
• Use a stereomicroscope to locate the DRGs after the spinal cord has been removed (Step 17A).
• Gently lift the spinal cord with forceps while performing the laminectomy to improve visualization of the DRGs as you progress along the thoracic and lumbar regions of the spine. If DRGs are clearly visible in the upper spinal segments, they should be identifiable in the L4-L5 segments (Step 15G).

### Problem 3: Poor immunocytochemistry

Clear staining of axons and neuronal somas is required for accurate quantification of axon outgrowth. If immunostaining produces high background signal or low contrast, it can be difficult to distinguish axons from the background during image analysis. Detection of specific proteins of interest may also require additional optimization of staining conditions.

### Potential solution

• Optimize antibody concentrations for the specific antibody being used. Blocking conditions, antibody concentration, incubation temperature, and incubation duration can all significantly influence antibody binding and specificity (Step 37 and 38).
• Protect immunofluorescent samples from light exposure during staining and storage.

### Problem 4: Conditioning effect is weak or absent

The conditioning effect in DRG neurons is time-dependent.^11^ Depending on the time point after injury, there may be variability in the extent of the conditioning effect. Additionally, if the nerve is not fully transected, fewer axons will be affected by the injury. Coverslip coating conditions can also significantly influence neuronal adherence and growth.

### Potential solution

• Ensure full transection of the nerve. The sciatic nerve has two nerve fascicles that may separate during manipulation; ensure that both are fully transected (Step 6C).
• While signaling and transcriptional changes can begin as soon as 1 day post injury, the conditioning response in DRG neurons typically reaches its peak ∼3 days after injury.^11^ The conditioning response can still be detected for up to 7 days post injury.
• Ensure that coverslips are properly coated with the appropriate concentrations of poly-L-lysine and laminin for the recommended duration. Deviations in substrate concentrations or coating conditions can affect neuronal adhesion and axon outgrowth (Step 10B, Step 10C).

### Problem 5: Overlapping axon outgrowth

Axons from individual DRG neurons may overlap with those from neighbouring neurons in culture. This overlap makes it difficult to quantify axon outgrowth from individual neurons, as the origin of each axon cannot be clearly distinguished. As a result, some neurons with substantial outgrowth may need to be excluded because their axons cannot be accurately traced.

### Potential solution

• Use a cell counter to ensure plating at a known and tested density that allows neurons to grow effectively without excessive overlap (Step 30).
• Reduce the culture duration if axons become too long and begin to overlap extensively. While we recommend imaging cultures at 16 h, significant differences in axon outgrowth can still be detected as early as 12 h (Step 32).

## Resource availability

### Lead contact

Further information and requests for resources or reagents should be directed to and will be fulfilled by the lead contact, Brett J. Hilton (bhilton@icord.org).

### Technical contact

Technical questions on executing this protocol should be directed to and will be answered by the technical contact, Eliana Seburn (eseburn@student.ubc.ca).

### Materials availability

This study did not generate new or unique reagents.

### Data and code availability

The published article includes all data generated or analyzed during this study.

## Acknowledgments

This work was supported by the 10.13039/501100000038Natural Sciences and Engineering Research Council of Canada (RGPIN-2024-03986) and the 10.13039/501100000196Canada Foundation for Innovation (John R. Evans Leaders Fund 44220). This project was made possible by the Canada Brain Research Fund (10.13039/100010707CBRF), an innovative arrangement between the Government of Canada (through Health Canada) and the 10.13039/100009408Brain Canada Foundation. E.S. is supported by a Canada Graduate Scholarship, Master’s from the 10.13039/501100000024Canadian Institutes of Health Research (CIHR). The graphical abstract was created with BioRender.

## Author contributions

E.S. designed the protocol, performed experiments, performed microscopy, analyzed data, generated the figures, and wrote the paper. B.J.H. supervised the study, designed the protocol, and edited the paper.

## Declaration of interests

The authors declare no competing interests.
